# Septic Shock Immediately following Percutaneous Suprapubic Catheterization

**DOI:** 10.1155/2021/2184866

**Published:** 2021-08-31

**Authors:** Cale E. Leeson, Brianna-Lee Beaudry, Geoffrey R. Wignall

**Affiliations:** ^1^Division of Urology, Department of Surgery, University of Ottawa, Ottawa, Ontario, Canada; ^2^Faculty of Medicine, Northern Ontario School of Medicine, Thunder Bay, Ontario, Canada; ^3^Division of Urology, Grey Bruce Health Services, Owen Sound, ON, Canada

## Abstract

Suprapubic catheterization (SPC) is considered a safe and effective procedure for long-term bladder decompression. With proper technique and appropriate patient selection, significant complications of SPC are rare. Immediate postoperative septic shock (i.e., within the first 24 hours of surgery) is rarely reported. We report a case of an 83-year-old patient who developed septic shock within one hour of suprapubic catheterization for a chronic hypotonic bladder, highlighting the importance of early recognition of complications from SPC and prompt management to ensure positive outcomes.

## 1. Introduction

Suprapubic catheterization (SPC) is widely considered a relatively safe procedure for both emergent and long-term bladder decompression. It is a management option for patients with neurogenic lower urinary tract dysfunction [1]. Compared to urethral catheterization, SPC may improve independence, reduce episodes of bacteriuria and pain, avoid the risk of urethral erosion, facilitate engagement in sexual activity, and decrease the risk of epididymitis in males [1, 2]. With proper technique and patient selection, major complications of SPC, such as bowel injury, are rare [2]. Immediate postoperative septic shock is rarely reported in the literature, though may be reduced when preoperative prophylactic antibiotics are administered in select patients [3]. We report a case of septic shock immediately following an elective percutaneous SPC in a patient with a chronic decompensated bladder.

## 2. Case Report

An 83-year-old female presented to the operating room to undergo an elective suprapubic catheter placement for chronic urinary retention secondary to a hypotonic bladder. Past medical history included hypertension, aortic stenosis, atrial fibrillation, transient ischemic attack, peripheral vascular disease, five vaginal deliveries, and longstanding pelvic floor dysfunction. The patient weighed 53 kilograms. Preoperative cystoscopy revealed a grossly trabeculated bladder consistent with long-standing urinary retention. A trial of clean intermittent catheterization was found undesirable by the patient, and a urethral catheter was placed. After discussion regarding long-term options, the patient decided to proceed with SPC.

In the operating room, cefazolin 1 g was administered intravenously on induction of procedural sedation. The bladder was distended with approximately 500 mL of normal saline instilled through a urethral catheter and adequately palpated. A spinal needle was used to infiltrate the tract with 0.5% Marcaine without epinephrine. The spinal needle was then advanced into the bladder, and urine was aspirated. A trocar was passed, and an 18 French Foley catheter was advanced into the bladder. Hematuria was noted, and continuous bladder irrigation (CBI) was started through the urethral catheter. The patient was transferred to the post anesthesia care unit (PACU) in stable condition. Within one hour of surgery, nausea, vomiting, rigors, pyrexia (temperature 39°C), and significant hypertension (229/165 mmHg) became apparent. On examination, the patient was alert and the abdomen was moderately distended and firm. Intravenous labetalol was administered, and a stat computed-tomography (CT) scan of the abdomen and pelvis was ordered to rule out bowel injury. Marked hypotension (82/50 mmHg) was then noted, and blood cultures as well as intravenous crystalloid resuscitation were ordered. The CT scan showed no evidence of bowel puncture and confirmed appropriate placement of the suprapubic catheter tip within the bladder.

The patient was then transferred to the intensive care unit (ICU) for management of postoperative septicemia. Fluid resuscitation and empirical antibiotics were administered until blood cultures and sensitivities were available. Vasopressors were required overnight and discontinued on postoperative day one, as was the CBI and the urethral catheter. The suprapubic catheter was well positioned and draining clear, yellow urine. White blood cell counts peaked at 30.6 × 10^9^/L. Blood cultures were positive for *Enterococcus faecalis* and *Proteus mirabilis*, for which the patient completed a seven-day course of piperacillin-tazobactam. *P. mirabilis* was sensitive to ampicillin, cefazolin, ciprofloxacin, gentamicin, and trimethoprim/sulfa, while *E. faecalis* showed sensitivity to ampicillin, gentamicin synergy, streptomycin synergy, and vancomycin.

The admission was further complicated by increased left ventricular afterload in the context of aortic stenosis, leading to poor coronary perfusion (postoperative troponin I peaking at 1917 ng/L) during the hypertensive episode in PACU. Coronary angiography was performed, and severe disease in the first obtuse marginal artery was treated with a drug-eluting stent. The patient was discharged in stable condition on postoperative day ten.

## 3. Discussion

Suprapubic catheters are indicated for chronic urinary retention in the elective setting [2]. Compared to urethral catheterization, SPC may reduce episodes of bacteriuria and pain and decrease the risk of epididymitis, prostatitis, and meatal erosion in males [1, 2, 4, 5]. Contraindications for SPC include patients presenting with carcinoma of the bladder or undiagnosed hematuria, sepsis of the abdominal wall, uncorrected bleeding disorders, or anticoagulation treatment or patients with a subcutaneous vascular graft in the suprapubic region [2]. Urologists should develop an individualized bladder management strategy for each patient [1].

SPC may be performed open or percutaneously, typically guided by ultrasound (US) or flexible cystoscopy [2]. The open technique is generally indicated for patients at high risk for bowel injury (previous lower abdominal surgery where the bladder has been mobilized or those in which the bladder cannot be distended sufficiently) [2]. US-guided SPC is recommended if the bladder cannot be sufficiently palpated, to avoid bowel injury [2]. The bladder should be adequately distended with at least 300 mL of irrigant to allow safe insertion, with larger volumes potentially increasing the margin of safety by providing a larger area of entry into the bladder [2]. In our case, the bladder was adequately distended and urine was aspirated to confirm placement. However, cystoscopy was not used to visualize for adequate distension, as the patient's urethral catheter was used to distend the bladder.

SPC is considered a relatively safe procedure for long-term bladder decompression. Complications include hematuria, catheter misplacement, surgical site infection, urinary tract infection, and bowel injury [2]. However, literature pertaining to immediate postoperative septic shock (i.e., within the first 24 hours) is limited. Two cases by Vaidyanathan et al. [6, 7] discuss fatal septicemia immediately following SPC for neurogenic bladder in patients with spinal cord injuries. They recommended that clinicians obtain a urine culture with appropriate antibiogram prior to cystostomy and avoid forcible distension of the bladder in patients with small bladder capacity or a colonized bladder from chronic indwelling urethral catheters [6, 7]. One study of 219 patients undergoing SPC found that 19% had complications at 30 days postoperatively, including 4.6% with septicemia secondary to a urinary tract infection (UTI) [3]. However, the study does not specify whether urosepsis occurred in the immediate postoperative period or whether septicemia secondary to UTI was merely associated with the presence of an indwelling suprapubic catheter. Bacteriuria will inevitably be present in any patient with an indwelling catheter and can result in symptomatic infection [2, 8]. Our patient developed sepsis within one hour of surgery. It is possible that the patient had a colonized bladder and low bladder volume, with rapid distension of the bladder during the procedure potentially contributing to the mechanism for the urosepsis. In a bladder with low compliance, acute distension can cause mucosal trauma facilitating entry of organisms present in the urine into the bloodstream, leading to bacteremia and, rarely, septicemia [7]. Acute distention of the bladder may also contribute to hematuria, such as in our case, from the overly stretched vesical mucosa [7]. Bladder distention has also been shown to significantly decrease coronary artery diameter at stenotic segments, leading to decreased coronary blood flow and increased coronary resistance [9]. This effect may have been compounded in our patient who had severe disease in the first obtuse marginal artery, contributing to the postoperative myocardial ischemia and subsequent rise in troponin.

As per current guidelines, our patient was managed for sepsis with adequate fluid resuscitation and vasopressors [10]. Microbiological cultures were obtained before promptly starting antimicrobial therapy [10]. Additionally, clinicians should have a low index of suspicion for bowel complications; thus, a rapid CT scan was ordered to rule out bowel injury [2]. Patients at high risk for infectious complications undergoing urologic procedures should receive preprocedural antibiotics [11]. Perioperative antimicrobial prophylaxis may reduce the risk of urosepsis following SPC [3]. Our patient had a chronic indwelling urethral catheter prior to undergoing SPC, increasing the risk for bladder colonization. Thus, cefazolin was administered intraoperatively, to which *P. mirabilis* was sensitive, though cefazolin may have been ineffective against *E. faecalis* based on postoperative culture and sensitivities. Despite this, our patient still developed septic shock, highlighting the importance of close monitoring following SPC in patients with chronic urethral catheterization for hypotonic bladder.

## 4. Conclusion

SPC is considered a safe and effective procedure for a decompensated bladder in appropriately selected patients. However, significant complications can arise. Preoperative urine cultures and antibiotic prophylaxis should be considered in patients who are high risk for bladder colonization, such as those with a chronic urethral urinary catheter. Our case highlights the importance of appropriate patient selection, risk factor identification, early recognition of complications from SPC, and prompt management to ensure positive outcomes.
